# Anglo-Swiss Milk Food

**Published:** 1884-05

**Authors:** 


### ANGLO-SWISS MILK-FOOD.

DECIDED superiority is claimed for the Anglo-Swiss Milk-Food in comparison with any other farinaceous food for infants. No so-called milk-food consists entirely of milk; all are partly composed of cereal products, involving, when not properly prepared, the presence of an injurious amount of starch, which the highest authorities agree in condemning for young children. The Anglo-Swiss Condensed Milk Company overcomes this objectionable feature of milk food, as usually supplied, by meeting an essential requirement in the method of preparing it, so that when gradually heated with water, according to the directions for use, the starch contained in the materials used is converted, in a satisfactory degree, into soluble and easily digestible dextrine and into sugar.

The Anglo-Swiss Milk-Food has beed found to meet these essential conditions to the satisfaction of physicians and others who have taken the pains to examine it, and we invite critical examination of it in comparison with any other food.

